# Hypercoagulopathy and Adipose Tissue Exacerbated Inflammation May Explain Higher Mortality in COVID-19 Patients With Obesity

**DOI:** 10.3389/fendo.2020.00530

**Published:** 2020-07-28

**Authors:** Gabriel Pasquarelli-do-Nascimento, Heloísa Antoniella Braz-de-Melo, Sara Socorro Faria, Igor de Oliveira Santos, Gary P. Kobinger, Kelly Grace Magalhães

**Affiliations:** ^1^Laboratory of Immunology and Inflammation, Department of Cell Biology, University of Brasilia, Brasilia, Brazil; ^2^Département de Microbiologie-Infectiologie et d'Immunologie, Université Laval, Quebec City, QC, Canada; ^3^Centre de Recherche en Infectiologie du CHU de Québec - Université Laval, Quebec City, QC, Canada

**Keywords:** adipose tissue, COVID-19, Obesity, SARS-CoV-2, hypercoagulopathy, ACE-2

## Abstract

COVID-19, caused by SARS-CoV-2, is characterized by pneumonia, lymphopenia, exhausted lymphocytes and a cytokine storm. Several reports from around the world have identified obesity and severe obesity as one of the strongest risk factors for COVID-19 hospitalization and mechanical ventilation. Moreover, countries with greater obesity prevalence have a higher morbidity and mortality risk of developing serious outcomes from COVID-19. The understanding of how this increased susceptibility of the people with obesity to develop severe forms of the SARS-CoV-2 infection occurs is crucial for implementing appropriate public health and therapeutic strategies to avoid COVID-19 severe symptoms and complications in people living with obesity. We hypothesize here that increased ACE2 expression in adipose tissue displayed by people with obesity may increase SARS-CoV-2 infection and accessibility to this tissue. Individuals with obesity have increased white adipose tissue, which may act as a reservoir for a more extensive viral spread with increased shedding, immune activation and pro-inflammatory cytokine amplification. Here we discuss how obesity is related to a pro-inflammatory and metabolic dysregulation, increased SARS-CoV-2 host cell entry in adipose tissue and induction of hypercoagulopathy, leading people with obesity to develop severe forms of COVID-19 and also death. Taken together, it may be crucial to better explore the role of visceral adipose tissue in the inflammatory response to SARS-CoV-2 infection and investigate the potential therapeutic effect of using specific target anti-inflammatories (canakinumab or anakinra for IL-1β inhibition; anti-IL-6 antibodies for IL-6 inhibition), anticoagulant or anti-diabetic drugs in COVID-19 treatment of people with obesity. Defining the immunopathological changes in COVID-19 patients with obesity can provide prominent targets for drug discovery and clinical management improvement.

## Introduction

On December 2019, a series of pneumonia cases without a recognized etiology was reported in Wuhan, a central China city (1). Rapidly spreading throughout the globe, Coronavirus disease (COVID-19) was recently discovered to be caused by Severe Acute Respiratory Syndrome Coronavirus 2 (SARS-CoV-2). The Word Health Organization (WHO) declared SARS-CoV-2 an international public health emergency on January 2020 and pandemic on March 2020. On June 25, 2020, ~9,527,124 COVID-19 cases were confirmed in the world and 484,972 deaths were considered to be caused by this disease. As a means of decelerating disease progression, health authorities advise their citizens to wear masks, wash hands (2) and increase in public and physical distancing (3).

COVID-19 symptoms may or may not include fever, fatigue, dry cough, dyspnoea, anosmia, dysgeusia, and diarrhea (4–6). Respiratory symptoms are believed to be caused by the occurrence of diffuse alveolar damage, tissue fibrosis, and chronic inflammatory infiltrates (7). Some of the COVID-19 patients can often also present with prominent changes in coagulation function (8). Common comorbidities observed in COVID-19 patients are hypertension, cardiovascular disease, type 2 diabetes (9), chronic obstructive pulmonary disease (10), and obesity (11).

Obesity is a major public health issue globally affecting a half a million people (12). As an inducer of cardiometabolic dysfunction, obesity is associated with increased risk for many diseases, such as Type 2 Diabetes (T2D) (13), dyslipidemia (14), hypertension (12), coronary disease (15), and coagulopathy (16). Chronic inflammation, defined as a low grade but persistent process, disrupting homeostasis and driving organ dysfunction (17). During obesity, chronic inflammation is not only associated with metabolic disturbances and decrease in heart health, but also impacts immune system function (18, 19). In this review we present evidence that indicates that people with obesity are more susceptible to develop severe forms of COVID-19 and higher mortality due to intrinsic alterations in blood coagulation parameters, inflammation, and immune response.

## Incidence of COVID-19 in People With Obesity

Obesity represents a risk factor for many chronic diseases, including hypertension, dyslipidemia, diabetes mellitus type 2 (T2DM), cardiovascular disease (20), and several types of cancer (21). As a consequence of excessive or abnormal fat tissue accumulation, overweight and obesity can alter innate and adaptive immune responses, making the immune system more prone to infections and less responsive to vaccinations, antivirals, and antimicrobial drugs (22). There is growing evidence that implicates obesity as one of the main risk factors for triggering severe forms of COVID-19 and poor outcomes (23).

Several studies have reported that obesity may affect the severity of COVID-19, with a direct correlation between increasing BMI and the proportion of patients with severe COVID-19 (24). It has been reported that comorbidities related to obesity are also correlated with increased COVID-19 mortality and morbidity, such as cardiovascular disease (22.7%), hypertension (39.7%), diabetes (19.7%), respiratory disease (7.9%), and cancers (1.5%) (25).

Several reports have shown a significant incidence of people with obesity presenting higher COVID-19 mortality and morbidity in different countries. According to WHO data, the United States of America ranks first in the world in terms of prevalence of obesity (36.2%), overweight (31.7%), as well as in the number of total deaths from COVID-19. Some American studies have indicated obesity as as important comorbidity deeply related to the development of severe forms of COVID-19 (26, 27). In a study developed in a large academic hospital in New York City investigating 3,615 individuals who tested positive for COVID-19, 775 (21%) had BMI values among 30–34 kg/m^2^ and 595 patients (16% of the cohort) displayed BMI values higher than 35 kg/m^2^ (28). Diabetes and obesity also increased the risk of COVID-19 infection in Mexico (29). In a French hospital that evaluated 124 patients admitted to intensive care by COVID-19, it was found that 28.2% of the cases had a BMI > 35 kg/m^2^ and required invasive mechanical ventilation (30). In Spain, from 48 critically ill COVID-19 patients admitted to ICU, 48% presented obesity and 44% arterial hypertension as most prevalent comorbidities (31). In Italy, the severity of COVID-19 and the tension in the health system related to the disease have been remarkable, with an estimated fatality rate of 7.2% (32). Recent Italian studies have highlighted the role of comorbidities in their COVID-19 cases, underlying obesity in the severity of this disease (33). Despite the low prevalence of obesity in China, the severely ill COVID-19 patients were older and had comorbidities, such as obesity and diabetes mellitus more often than non-severely ill individuals (34). In Republic of Korea, clinical data of COVID-19 early cases were collected and demonstrated that of the 28 hospitalized patients, 17.9% had one or more coexisting medical conditions being obesity the most common comorbidity (35).

Therefore, considering that obesity is one of the strongest risk factors for COVID-19 severity, it is important to better understand the correlation between obesity and COVID-19 and the mechanisms that could be involved in this process. In this way, it is crucial to analyze deeper this issue, focusing on the association among SARS-CoV-2 host cell entry, adipose tissue biology, and all the inflammatory, vascular and metabolic dysfunctions that may define COVID-19 progression.

## SARS-CoV-2 Host Cell Entry

Genomic analyses demonstrated that SARS-CoV-2 is 96% identical at the whole-genome level to a bat coronavirus SARS-CoV (36). It has been demonstrated that host cell entry of SARS-CoV-2 depends on the same receptor used by SARS-CoV to entry host cell, the Angiotensin-converting enzyme 2 (ACE2) (36, 37). ACE2 receptor was first described in 2000 (38, 39) and it was associated with multiple pathophysiological processes, including the pathogenesis of cardiovascular and renal diseases such as hypertension, myocardial infarction and heart failure (40), acute lung injury (ALI) (41), and acute respiratory distress syndrome (ARDS) (42, 43).

ACE2 gene contains 18 exons and 20 introns, maps to Xp22 chromosome and spans 40 kb of the genomic DNA (44). ACE2 protein is a type I transmembrane glycoprotein of 805 amino acids (~120 kDa), containing a single extracellular catalytic domain whose sequence is 41.8% identical with the domain of angiotensin-converting enzyme (ACE) (43). ACE2 is part of the renin-angiotensin-aldosterone system (RAAS), which is a peptidergic system that acts in the homeostatic regulation of the renal and cardiovascular systems, regulating extracellular fluid volume (40). Renin (an aspartyl proteinase secreted by kidney into the circulation) cleaves its starting substrate angiotensinogen to angiotensin I, which is hydrolyzed by ACE to angiotensin II. ACE2 cleaves angiotensin II to Angiotensin 1-7. Angiotensin II promotes inflammation, oxidative stress, vasoconstriction, salt and water reabsorption (45). Consequently, increased ACE2 activity can shift the balance to the Angiotensin 1-7 axis, leading to disease and inflammation protection. Moreover, ACE2 is a zinc metalloprotease multifunctional enzyme that can act on several vasoactive peptides (46), regulating important cardiovascular and renal functions. Therefore, ACE2 has an ambiguous role acting as both an important physiological receptor and a SARS-CoV-2 backdoor (47).

SARS-CoV-2 bind to its host cell receptor is a critical initial step for this virus entry into target cells. SARS-CoV-2 use the homotrimeric spike glycoprotein S on the viral envelope to bind their cellular receptors, which facilitates viral attachment to the surface of target cells, inducing endocytosis of virion particle, catalyzing the fusion between viral and host cell membranes, and allowing the entry of the virus genome into the host cell cytoplasm. Each monomer of trimeric S protein is about 180 kDa, and contains two subunits, S1 and S2. S1 mediates viral attachment to host cell and S2 intermediates membrane fusion.

**Figure 1:**
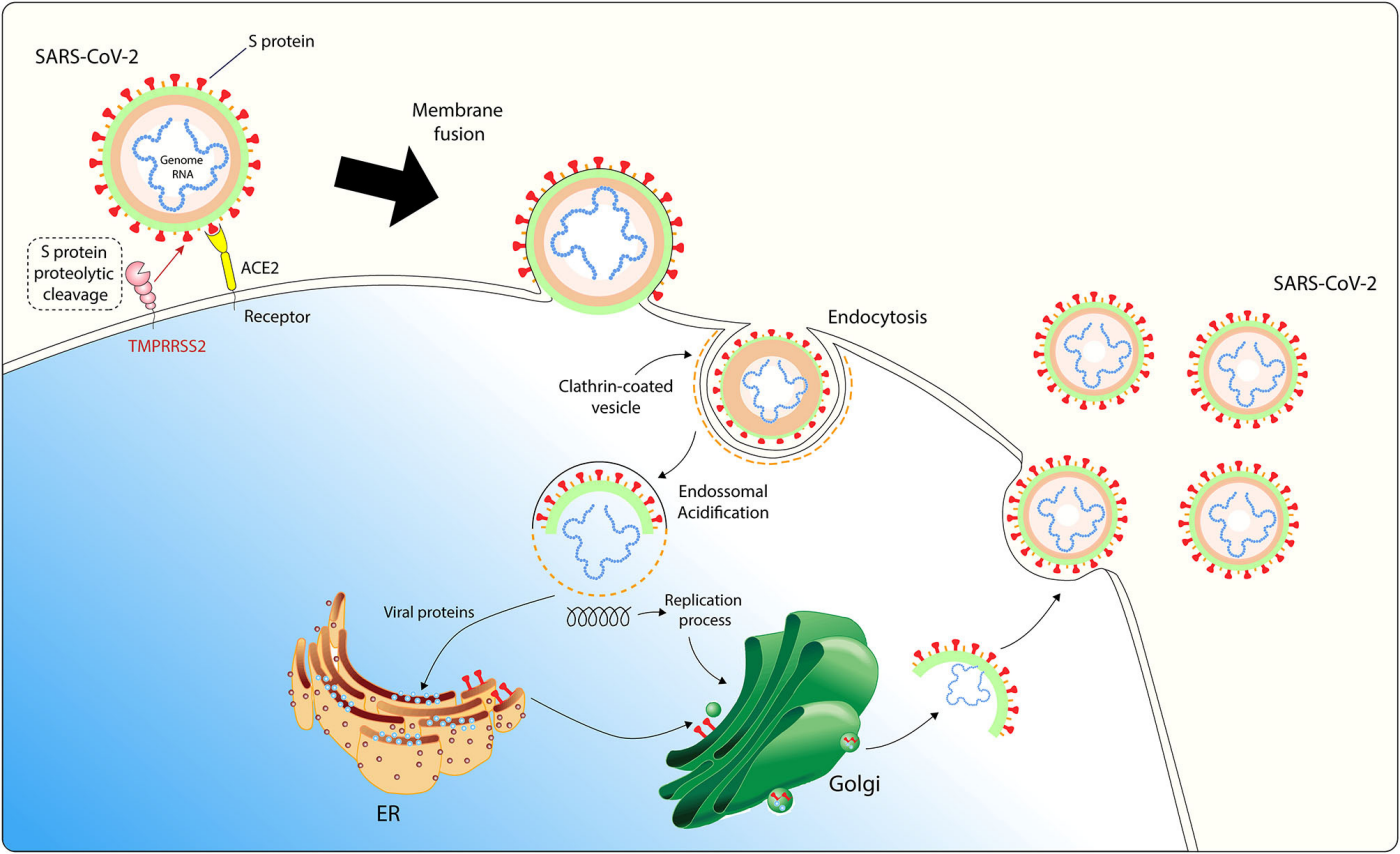
SARS-CoV-2 host cell entry cell depends on the angiotensin-converting enzyme 2 (ACE2) and TMPRSS2. The virus uses the homotrimeric peak glycoprotein S present on viral envelope surface to physically interact with its cell receptor, which facilitates binding to the surface of target cells, enables endocytosis of the virion particle and entry of the viral genome into the host cell cytoplasm. This cell entry process requires priming of protein S by cellular proteases, such as the serine protease TMPRSS2. After endosomal acidification, viral proteins are synthesized in host ER for viral replication and virion particles shedding occurs through Golgi apparatus. ACE2 is expressed in adipocytes and adipose tissue may act as a reservoir for SARS-CoV-2.

SARS-CoV-2 entry in host cell requires S protein priming by cellular proteases, such as the endosomal cysteine proteases cathepsin B and L (CatB/L) and the cellular and the serine protease TMPRSS2 (37). SARS-CoV-2 entry into susceptible cells is a complex process that requires the combined action of receptor-binding and proteolytic processing of the S protein to promote an efficient virus-cell fusion (48), followed by endosomal acidification (Figure 1). Contrasting SARS-CoV, cells infected with SARS-CoV-2 form typical syncytium, suggesting that SARS-CoV-2 may mainly use the plasma membrane fusion pathway to enter and replicate inside host cells (49). This plasma membrane fusion pathway is more efficient for most viruses since it may delay host cell antiviral immunity activation compared to the viral and endosomal membrane fusion pathway (50, 51).

Considering the recent findings showing that SARS-CoV-2 mainly uses TMPRSS2 for plasma membrane fusion, clinically proven inhibitors of the cellular serine protease TMPRSS2 might constitute an option for blocking SARS-CoV-2 host cell membrane entry. These results have important implications for our understanding of SARS-CoV-2 transmissibility and pathogenesis and reveal a target for therapeutic intervention.

Several studies have demonstrated how SARS-CoV-2 uses the human ACE2 as the main receptor to viral entry into host cell (36, 48, 52). Overexpression of human ACE2 led to more severe disease in a mouse model of SARS-CoV infection, indicating that viral entry into host cells is a key step for the establishment and progression of this disease (53). Moreover, Zhou and colleagues demonstrated that overexpressing human ACE2 in HeLa cells allowed increased SARS-CoV-2 infection and replication (36). ACE2-expressing cells may act as target cells and are susceptible to SARS-CoV-2 infection (54) and S protein-targeted neutralizing antibody may be prominent antiviral tools against SARS-CoV-2 infection. Several cellular types have been identified with high ACE2 expression including myocardial cells, type II alveolar cells, proximal tubule cells of the kidney, ileum and esophagus epithelial cells, and bladder urothelial cells (54), epithelial cells of oral mucosa (55), nasal epithelial cells (56), and interestingly, adipocytes (57).

## SARS-CoV-2 and Adipose Tissue

In patients with obesity, in which white adipose tissue (WAT) is exacerbated and brown adipose tissue (BAT) is decreased (58), RAAS is chronically activated and predisposes the individual to a plethora of dysfunctions, including heart and kidney pathologies. These alterations are associated not only with high blood pressure (59) but also related to insulin signaling in peripheral tissues (60), inflammatory status in pancreas and death profile of β cells (61). Increased oxidative stress is believed to be the root of the cytotoxic effects induced by Ang II and aldosterone during RAAS aberrant activation (62). The resulting insulin resistance acts as a driving force for the progression of cardiometabolic syndrome, commonly associated with obesity (63).

The counterbalance for this blood pressure-increasing action of RAAS is the alternative pathway, which consists in the agonism of the G-protein-coupled Mas receptor by Ang-(1-7). This compound is generated by the enzymatic activity of ACE2 in both AngI and AngII. Ang-(1-7) induces vasorelaxation and cardioprotection (64). ACE2/Mas axis induction associates with BAT activation and WAT browning, processes that are related to anti-obesity effects (65). Due to many alterations in the physiology during obesity, including RAAS dysfunction, BAT tend to present decreased size and activation, increasing the chance of comorbidities (58).

RAAS components, including ACE2, are expressed in adipocytes and are crucial for their glucose and lipid metabolism homeostasis (63). *In vivo* experiments in mice showed that high-fat diet (HFD)-induced obesity is associated with increased adipose tissue ACE2 expression (40). Thus, we hypothesize here that increased ACE2 expression in adipose tissue displayed by people with obesity may increase SARS-CoV-2 infection and accessibility to this tissue. Moreover, obesity causes hyperglycemia via insulin resistance whereas growing evidence demonstrates that SARS-CoV-2 may cause hyperglycemia as well by infecting and killing B-cells (66). In addition, some drugs often used for the treatment of patients with obesity complications (such as antihypertensives, statins, thiazolidinediones) can up-regulate ACE2, thus could potentially increase the viral up-take (67–70).

Obesity is characterized by dysfunction of immune system (19). During obesity, systemic inflammatory status is influenced by intense pro-inflammatory cytokines secretion (71), increasing the chance of cytokine storm occurrence (72). In addition, obesity associates with Type I Interferon (IFN) decreased secretion, key players in antiviral immune response (73). Human studies showed that H1N1-infected patients with obesity stayed longer under ICU care (74), and investigations with Diet-induced obese (DIO) murine model informed that over nutrition impaired antiviral response against Influenza (75). Thus, individuals with obesity present immune system dysfunction that increase respiratory viral infection susceptibility (19).

It is currently known that adipocytes (76), which are the main cellular components of adipose tissue, and lung cells are targets for SARS-CoV-2 infection (77). Influenza A also presents shared tropism for lungs (78) and WAT (76). In an elegant study, Maier and colleagues showed that symptomatic adults with obesity shed influenza A virus more than 40% longer than non-obese adults. They suggested that WAT dysregulation, common in individuals with obesity, is related to prolonged viral shedding duration (76).

The alarming COVID-19 morbidity and mortality rates of individuals affected with heart pathologies may be related to epicardial adipose tissue (EAT). Classified as a visceral AT, EAT may act as a SARS-CoV-2 reservoir, prolonging viral shedding to cardiac tissue. In addition, EAT obtained from subjects with obesity tend to present higher levels of IL-6 and TNF-α (79), cytokines abundantly secreted in COVID-19 patients. Furthermore, the ACE2/Mas axis dysfunction, observed in individuals with obesity and in subjects affected by COVID-19, associates with EAT inflammation, probably due to Ang(1-7) level decrease, once this protein is associated with diminished proinflammatory macrophage polarization in EAT (80). Once metabolic syndrome, common in individuals affected by obesity, is associated with increased amounts of EAT (81), alterations in EAT amount and inflammatory status may be suggested to influence COVID-19 cardiac morbidity in individuals living with obesity. EAT measurement may play a crucial role in the management of COVID-19 progression in cardiac patients (82).

In a study investigating the influence of obesity in the prognosis of asthma patients, Elliot and others showed that individuals affected by obesity display WAT deposits in large airway walls. They found that BMI value impacts proportionally WAT deposits size, which favors both airway wall thickness increase and neutrophil infiltration within pulmonary tissue (83). Increase in lung wall thickness associates with difficulties in gas exchange (84), and immune cells infiltration is related to tissue damage and fibrosis (85). It is important to have in mind that the increased expression of ACE2 in WAT during obesity makes these intra-pulmonary deposits a susceptible point for SARS-CoV-2 infection within the lung tissue. In addition, the prolonged viral shedding that may occur in WAT would facilitate for the occurrence of pulmonary damage and consequent respiratory failure in cases of obesity (76).

Also found in lungs, adipose-like cells called lipofibroblasts (LiFs) affect pulmonary function, since the transdifferentiation of these cells to myofibroblasts leads to pulmonary fibrosis (PF) (86). LiFs present lipid droplets (LDs) within their cytoplasm containing high levels of perilipin-2. Located in the alveolar interstitium, these cells reside in the proximity of ACE2-expressing type 2 alveolar epithelial cells (AEC2), to whom they provide surfactant molecules. AEC2 are considered to be the biggest pool of ACE2-expressing cells in the lungs and LiFs proximity may indicate higher chance of PF in the lungs of infected individuals with obesity (87). In addition, the possibility of LiFs to also express ACE2 should be assessed, once PF is a common feature among deceased COVID-19 patients.

Although WAT dysfunction is associated with high rates of COVID-19 morbidity and mortality in individuals with obesity, WAT can be a promising source of mesenchymal stem cells (MSCs). As described by Leng and others, intravenous administration of clinical-grade MSCs was capable of improving pulmonary functional activity into seven COVID-19 patients (88). Due to its accessibility and amount of stem cells, subcutaneous WAT (scWAT) is the main source of MSCs, the AT-derived stem cells (ASCs). Once ASCs display high secretory activity, they possess therapeutic potential for the treatment of pulmonary damage caused by COVID-19 (89).

**Figure 2:**
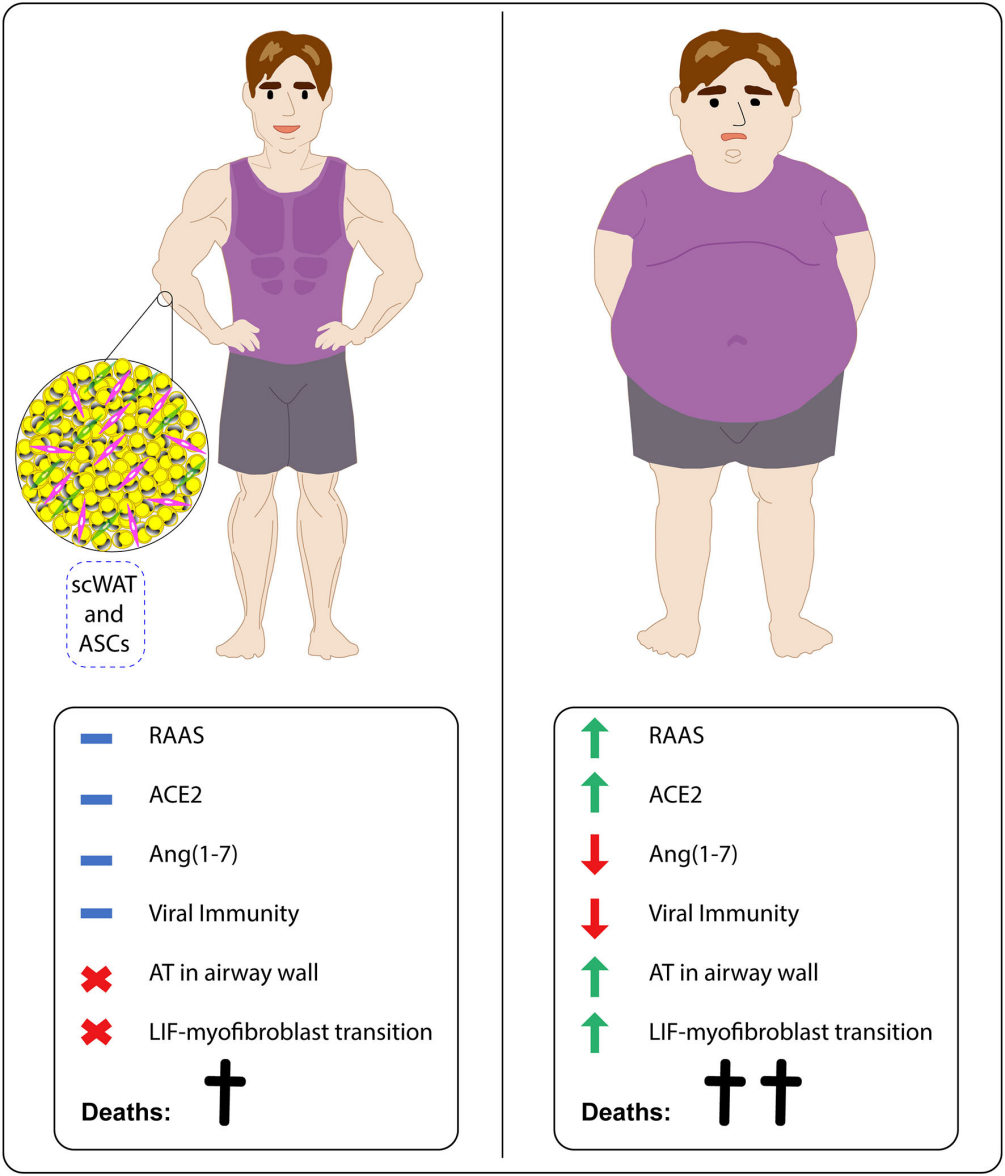
Obesity increases SARS-CoV-2 infection vulnerability in affected individuals by interfering in RAAS activation, antiviral immunity, fat tissue accumulation, and differentiation status of pulmonary fibrosis related cells. While lean individuals tend to show adequate RAAS activation, including ACE2 and Ang(1-7) levels, effective antiviral immune responses and absence of both adipose tissue (AT) deposits in airways and LiF-myofibroblast transdifferentiation, subjects with obesity display aberrant RAAS activation, favoring high ACE2 expression and low Ang(1-7) availability, decrease of immune responses against viruses and presence of both AT deposits in airways and LiF-myofibroblast transdifferentiation. ScWAT-derived stem cells (ASCs) could be applied in COVID-19 treatment.

Therefore, we suggest that individuals with obesity tend to be more susceptible to SARS-CoV-2 infection and COVID-19 progression. These patients show aberrant RAAS activation, high ACE2 levels, low Ang(1-7) amounts, decreased antiviral immunity, higher amounts of EAT and presence of lipid deposits in large airways, which potentially act as viral reservoirs in heart and lung proximities, and higher chances of LiF-myofibroblast transdifferentiation and consequent pulmonary fibrosis. These features help to explain the disturbing statistics related to susceptibility, morbidity, and mortality of individuals affected with obesity. Research under potential applicability of ASCs is crucial for alleviating the impact of SARS-CoV-2 infection on this risk group (Figure 2).

## Inflammatory Alterations in Obesity and COVID-19

Exacerbated inflammation is associated with increased risk of severe disease and mortality in patients with COVID-19 (90). COVID-19 patients commonly present intense pro-inflammatory markers activation such as IL-1, IL-6, IL-17, IL-18, IFN, and C-reactive protein (90, 91) with deep lymphopenia and substantial mononuclear cell infiltration in the lungs, heart, lymph nodes, spleen, and kidney (92, 93). Considering that the mortality and morbidity observed in COVID-19 patients is associated with excessive inflammation, a better understanding of the immunological parameters seen in patients infected with SARS-CoV-2 and people with obesity is necessary to better correlate COVID-19 and obesity, improving the identification of therapeutic targets.

Inflammation is an essential factor for the protection against countless threats that affects the organism during a lifetime. Deficiencies on immune system activation arises several disorders, which can be deleterious depending on the immunosuppressive potential of the disease (94). On the other hand, the chronic or excessive activation of the immune system also contributes to homeostasis breakdown and play a key role on classic inflammatory diseases progression, such as obesity and other metabolic disorders (95, 96). Obesity has been characterized by low grade chronic inflammation. This process leads to exacerbated and prolonged activation of both innate and adaptive immune responses, bringing on tissue damage and metabolic and physiologic alterations.

WAT is the central organ that orchestrates obesity and is composed by different kind of adipocytes, immune and endothelium cells, among others. During obesity, pro-inflammatory cytokines are overexpressed concomitantly with adipocytes hypertrophy and hyperplasia (71). The uncontrolled increase in the number and content of adipocytes lead to hypoxic microenvironment that is associated to cellular necrosis, activating local immune response (97). In the WAT, immunological cells, such as macrophages, natural killers (NK), T and B lymphocytes are major sources of interleukin-6 (IL-6) and tumor necrosis factor-alpha (TNF-α), which are central cytokines on driving inflammation linked to comorbidities establishment (98–100). In addition, macrophages are recruited by increased expression of monocyte chemoattractant protein-1 (MCP-1) and polarized to their pro-inflammatory profile due to the abundance of IL-6 and TNF-α (101–103). The huge macrophage infiltrate in the adipose tissue that accompanies obesity increases the source of inflammatory mediators, thus maintaining a chronic and persistent inflammation that affects systemic metabolism and immune response (99).

In obesity, inflammatory markers are found altered not only in the adipose tissue, but also in the serum, liver, skeletal muscle, lung, among other organs (71, 104, 105). As a result, the impact of immunomodulatory potential of obesity compromises systemically the response against homeostasis breakdown. Some comorbidities, such as insulin resistance, T2DM, hypertension, pulmonary illness, fatty-liver, and cardiovascular diseases are direct related to the chronic inflammation provided by obesity (106–108). In addition, these inflammatory modulations alter how the organism will face different pathogens infections, leading obesity to be considered a risk factor of a great number of them.

The intensive secretion of IL-6, TNF-α, and MCP-1 sustains the unbalanced inflammation on individuals with obesity. Together, these inflammatory mediators lead to several alterations on systemic responsiveness to nosocomial infections, increasing the incidence of generalized inflammation by the cytokine storm release (109, 110). Moreover, population with obesity also maintains a chronic inflammation on respiratory tract, presenting a higher susceptibility for acute lung injury caused by viral infections, such as H1N1 (110, 111). However, despite intensive inflammation has been considered factor that plays a major role in the higher mortality of population with obesity on several viral illnesses (110), there are other alterations on immunological system that contribute to this scenario. Currently, it has been shown a downregulation of a central pathway during immune system activation against viral infection, being also a factor responsible for the greater involvement observed during obesity (73).

Studies have shown impairment of IFN secretion on individuals with obesity, besides other pro-inflammatory cytokines are being overly produced (73, 112). IFN is the most important cytokine for combating viral infections and reducing this signalization pathway makes the organism more susceptible to the severity of viral disease (113, 114). Moreover, influenza vaccination seems to have a worse performance on obese or overweighed population, demonstrating that the efficacy of adaptive immune response is also decreased (115, 116). During influenza vaccination in humans, type I IFN signalization has been shown to be modulated throughout dendritic cells activation, which is central for long-term CD8+ T cells immunity (117). In addition, the use of type I and III IFN as vaccine adjuvants have demonstrated benefits for adaptive immune response development *in vivo* (118). The leptin overproduction found in individuals with obesity leads to an aberrant type I interferon secretion, thus impacting how the organism will handle vaccination-induced immunity (112). Currently, it is not possible to affirm if SARS-CoV-2 vaccine response will be less effective on individuals with obesity. However, the available data regarding the immune response of obese or overweighted individuals *in vivo* demonstrate that hiporresponsiveness to a COVID-19 vaccine could be a concern (119–121).

Thereby, it is clear that therapeutic targets may not be focused on turning off the pan activation of the immune system, but through the induction of the appropriate mediators for better combating the infectious agent. Moreover, the maintenance of controlled levels of inflammatory mediators is essential for homeostasis establishment and tissue recovery. Therefore, therapeutic targets aiming inflammatory response may be proposed to improve treatment of COVID-19 patients with obesity, once this group is at higher risk for developing severe viral illness considering their intrinsic alterations in inflammatory profile.

## Coagulation Alterations in Obesity and COVID-19

Alterations in blood coagulation parameters have been increasingly implicated in COVID-19 severity, mortality, and morbidity (122–124). Several recent studies have shown that COVID-19 is commonly complicated with coagulopathy and disseminated intravascular coagulation (DIC) or associated with hypercoagulability together with a severe inflammatory state (125), leading to higher mortality (1, 8, 126). COVID-19 patients with acute respiratory failure present a severe hypercoagulability rather than consumptive coagulopathy (127). A significant portion of the patients hospitalized with COVID-19 usually present a pattern of coagulopathy characterized by elevations in D-dimer levels (8) and fibrin/fibrinogen degradation products, while abnormalities in prothrombin time, partial thromboplastin time, and platelet counts are relatively uncommon in initial presentations (123). Indeed, the DIC seen in the COVID-19 infection is clinically evidenced with high concentrations of D-dimer, being a poor prognostic characteristic (128) and higher risk of mortality (9). COVID-19 patients show a fulminant activation of coagulation and consumption of coagulation factors, with severe thrombocytopenia (low platelet count) (129). Likewise, obesity is highly related to a hypercoagulopathy status.

Excess body weight and especially abdominal fat accumulation can increase cardiovascular diseases morbidity and mortality, directly and indirectly. Direct effects are mediated by the structural and functional adaptations of the cardiovascular system to accommodate excess body weight, as well as by adipokine effects on inflammation and vascular homeostasis, leading to a pro-inflammatory and pro-thrombotic state. Indirect mechanisms occur concomitantly to other factors, such as insulin resistance, T2DM, visceral adiposity, hypertension, and dyslipidemia (130, 131).

Apart from metabolic and hemodynamic changes, central adiposity is also characterized by a systemic oxidative stress process, leading to the loss of the antithrombotic properties of endothelium (132). This mechanism partially supports the obesity as a pro-thrombotic clinical condition, presenting increased platelet activation and decreased fibrinolysis (133). Stimulation of vascular endothelium, platelets, and other circulating vascular cells by exacerbated production of proinflammatory cytokines by people with obesity promotes the upregulation of procoagulant factors and adhesion molecules, downregulation of anticoagulant regulatory proteins, increased thrombin generation, and enhanced platelet activation (134).

**Figure 3:**
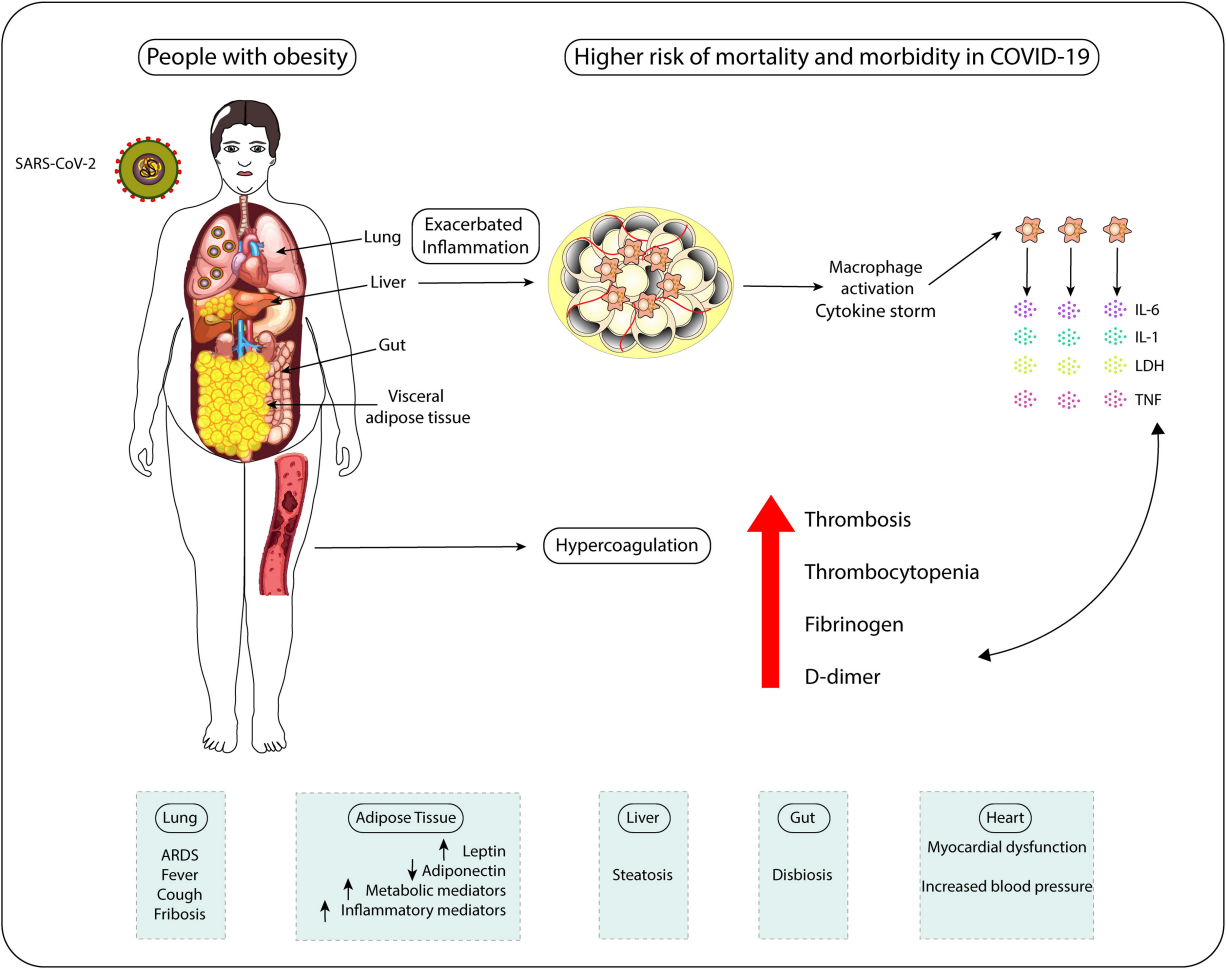
People with obesity display higher risk of mortality and morbidity in COVID-19 due to exacerbated inflammatory status and hypercoagulation tendency. Individuals with obesity show systemic chronic inflammation, which favors macrophage activation, cytokine storm occurrence (aberrant secretion of pro-inflammatory cytokines IL-6, IL-1, and TNF) and cytotoxicity (LDH release). This inflamed status associates with the increased clotting risk (hypercoagulation) presented by these patients. All these features make subjects with obesity more prone to develop pathological alterations in the physiology of lungs, AT, liver, heart, and intestines, which negatively influences gut microbiome composition. The impact of obesity-associated chronic inflammation on systems' physiology, including on antiviral immune responses, and the increased levels of coagulation-inducing mediators (fibrinogen and D-dimer) in COVID-19 patients help to explain the higher risks of these individuals to die of COVID-19 and to suffer with this infection compared to non-obese individuals.

Adipose tissue could play a crucial role in the induction of a procoagulant state in obesity. Obesity is associated with overproduction of procoagulant microparticles (MP) and increased Tissue factor (TF), a primary initiator of the blood coagulation cascade through its Factor VII receptor activity, leading to hypercoagulopathy (135, 136). Moreover, release of adipokines/inflammatory factors by adipose tissue, such as TNF-α, IL-8, and IL-6, can lead to the release of ville Willebrand factor (vWF) from the endothelium and elevate platelet activation and aggregation, inducing coagulation factors production and changes in the vessel wall, thus contributing to the thrombosis event (137). Platelets store cytokines and growth factors and the entire subcellular apparatus for protein synthesis involved in the coagulation cascade, IL-1β, plasminogen activator inhibitor-1 (PAI-1) and TF (138). The inflammatory effects of cytokines also result in endothelial injury (139) and the substantial increase in the production of pro-inflammatory cytokines results in a cytokine storm, leading to an elevated risk of vascular hyperpermeability, organ failure, and death (140). Moreover, the platelets of individuals with obesity exhibit a series of abnormalities contributing to the status of hypercoagulability observed in these people (141). In this way, an inherent exacerbated inflammation state and a tendency to develop hypercoagulation together are the main causes for people with obesity present higher mortality rates due to COVID-19 (Figure 3).

**Figure 4:**
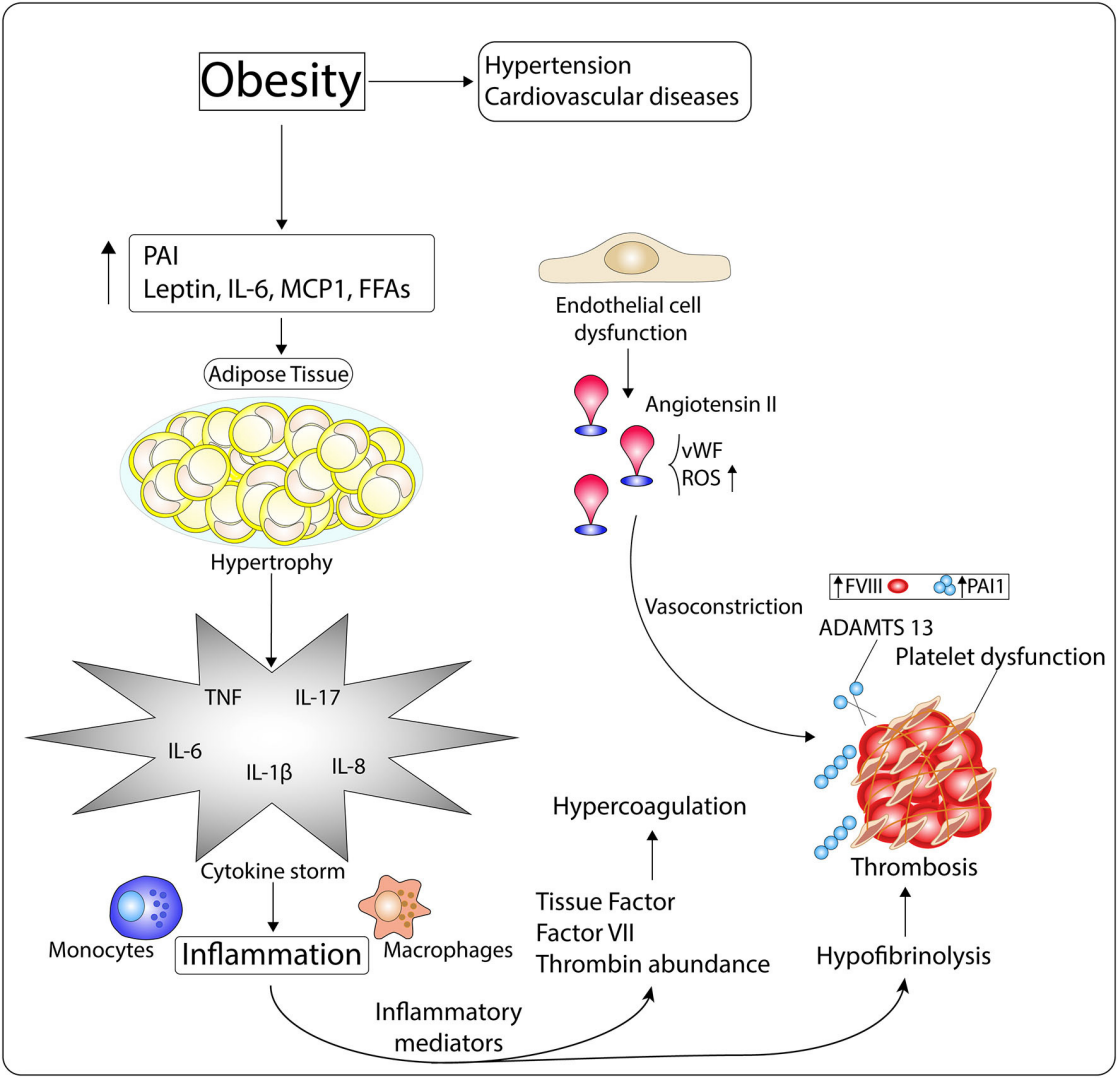
Mechanisms of the hypercoagulopathy and exacerbated inflammation observed in people with obesity. Obesity, which is intimately related with the pathogenesis of hypertension and cardiovascular diseases (CVDs), is characterized by high levels of Plasminogen Activator inhibitor I (PAI), leptin, IL-6, MCP-1, and free fatty acids (FFAs). These molecules induce white adipocyte hypertrophy, which then enables the occurrence of inflammation through cytokine storm. Inflammatory mediators impact on the availability of tissue factor (TF), factor VII (FVII), and thrombin-inhibiting factors, disrupting procoagulant-anticoagulant balance, and leading to hypercoagulation. Moreover, inflammation dysregulates fibrinolytic homeostasis through platelet dysfunction, increased FVIII and PAI-1 levels, and diminished ADMTS 13 activity, which enhance the risk of thrombosis. In addition, endothelial cell dysfunction, common in the obese phenotype, associates with Angiotensin II (Ang II) elevated amounts, von Willebrand factor (vWF) release, and oxidative stress, which induce vasoconstriction and thrombus formation.

It has also been shown that pro-trombotic factors are positively related to central fat. People with obesity have higher plasma concentrations of all pro-thrombotic factors (factor VII, fibrinogen, and vonWillebrand factor), as compared to non-obese individuals (142). Similarly, plasma concentrations of PAI-1, a physiological inhibitor of plasminogen activators (urokinase and tissue types) synthesized by adipose tissue, is highly elevated in plasma of people with obesity (143–145), predisposing those individuals to thrombotic complications. All of these conditions contribute to the progression of the prothrombotic state found in obesity (Figure 4).

Genetic factors are also correlated with higher susceptibility to coagulation impairment among individuals (146). Similarly, growing evidence has also supported the role of genetic factors as influencers in COVID-19 respiratory failure. In this context, a report has shown an association between ABO blood system and COVID-19 symptoms variation among individuals (147). This genome meta-analysis is in accordance with previous reports, in which blood-type O individuals are less affected by respiratory complications than type A (148, 149). These data are interesting, once blood-type O individuals are also less susceptible for thromboembolism development compared to non-type O (A, B, or AB) (150, 151). Until this moment, there is no evidence that obesity can be modulated by blood type. However, blood coagulation and COVID-19 severity need special attention since they are both influenced by ABO system. Understanding the inherent factors that support coagulation dysfunctions observed in COVID-19 patients is important to the establishment of therapeutics against this disease.

The impact of obesity, often related with other comorbidities, has been highlighted in severe forms of COVID-19 (152). In critically ill patients, coagulation alterations and inflammation are observed, with increased D-dimer and fibrinogen levels and, consequently, associated with a worse prognosis (126). The inflammation and hypoxemia related with a prothrombotic state are significant features of severe forms of COVID-19.

In conditions of obesity, coagulation disorders are emerging as an important issue in SARS-CoV-2 infection. Activation of leukocytes, endothelial cells, and platelets through the cytokine storm, positive regulation of TF and the subsequent generation of thrombin and fibrin formation can mediate the metabolic and cardiovascular complications associated to obesity (153). Together with coagulopathy, the thrombus formation in the microvascular environment contributes to tissue ischemia and organ dysfunction (154).

Under physiological conditions, shear stress increases the expression of ACE2, promoting the production of nitric oxide and reducing inflammation and proliferation in vascular endothelial cells. Endothelial cell activation/damage due to the coronavirus binding to the ACE2 receptor promoting acute inflammation and hypercoagulation may be of importance to explain the thrombotic burden observed (155). Primarily, angiotensin II induces PAI-1 expression by endothelial cells via the AT1 receptor, giving to a PAI-1/tPA shortcomig and a hypercoagulable state (1). Additionally, angiotensin II stimulates PAI-1 release from adipocytes and may in part account for the increased severity observed in those individuals with high BMI (156).

Clinically, the most prominent coagulation marker is elevation of D-dimer levels that has been consistently reported in many studies, representing a prognostic indicator for severity and mortality of disease (157). The high D-dimer indicates that other inflammatory markers including ferritin, IL-6, troponin I and lactate dehydrogenase (LDH) are accompanied by a secondary hypercoagulable condition (9, 158). It also has been suggested that the sustained inflammation due presence of continuous consumptive coagulopathy may contribute to the thrombocytopenia (159).

Therefore, it is highly recommended that COVID-19 patients with obesity are early and rapidly tested for coagulation screening, including the measurement of D-dimer and fibrinogen levels, following thromboembolic prophylaxis for critically ill hospitalized patients. Moreover, anti-coagulation drugs, such as heparin, may be especially important for treatment of COVID-19 patients with obesity, potentially leading to lower mortality rates in this high susceptible group of individuals.

## Obesity and therapeutics against COVID-19

Obesity-related conditions increase the risk of disease severity caused by the SARS-CoV-2 (160, 161). Daily use of several medications is necessary for controlling such conditions and an important influence of them on the body's responsiveness to infections are being extensively discussed worldwide (162–165). Studies regarding this interaction create potential therapeutic targets aiming to soft or protect against the intense symptoms. In the absence of a COVID-19 vaccine, the study of available promising drugs is essential for combating the COVID-19, to reduce the high mortality rate observed on people with obesity.

As previously discussed, the chronic inflammation presented in the lung during obesity leads to a cytokine storm by overexpressing pro-inflammatory mediators, such as IL-6 and TNF-α (166). This is one of the mechanisms through which host unbalanced inflammation worsens the prognosis of individuals with obesity affected by COVID-19 (90, 167). Thereby, anti-inflammatory drugs could have an important role on protecting specially people with obesity against COVID-19 multi-organs damage, once inflammation is an inherent characteristic of obesity and COVID-19 aggravation. However, this has to be carefully analyzed, once reducing pan inflammatory response can also prolong the time required for effective virus clearance (167).

Effective class of anti-inflammatory drugs against COVID-19 is a concern. It has been suggested an increasing risk for developing severe COVID-19 by the use of non-steroidal anti-inflammatory drugs (NSAIDs) and corticosteroids. The chronic use of NSAIDs is associated to increased cardiovascular and pulmonary outcomes (168, 169), complications which are also found in COVID-19. Considering this, a synergism can in fact occur, but no evidence regarding this interaction to SARS-CoV-2 is available yet (170). Corticosteroids anti-inflammatory drugs lead to a high suppression of innate immune system and delay of viral clearance (171). Harmful responses have already been found after corticosteroids medication for different respiratory virus infection, such as influenza, SARS-CoV and MERS-CoV (165, 172). Nevertheless, the use of this class of drugs seems to have no positive interference on COVID-19 cases (173, 174). However, a preliminary *in vitro* study showed SARS-CoV-2 replication suppression by the use of a corticosteroid (175). Moreover, a correlation between lower gene expression of ACE2 and TMPRSS2 with inhaled corticosteroids medication in asthma patients has already been demonstrated (176). The current scenario of available data indicates that consistent evidences about benefits or harms of corticosteroids use on COVID-19 still need deep investigation for accurate conclusions. In the absence of both positive and negative conclusion about this question, clinicians are avoiding to prescribe this class of pan anti-inflammatory drug for COVID-19 cases, since controversial reports make the conclusion about their real impact still unknown.

Considering the above mentioned points, reports have suggested that specific cytokine blocking could be more efficient for protection against COVID-19 than systemic anti-inflammatory drugs (165, 177). In this context, the use of monoclonal antibodies makes it possible to selectively inhibit key agents that drive to hyperinflammation during COVID-19. Preliminary evidence suggests that IL-6 blockade could be helpful on curbing the cytokine storm, being a highly promising treatment for severe COVID-19 (178). Nevertheless, the clinical trials that could provide trustable answer about this question are still in progress (179, 180). In addition, studies regarding TNF-α therapy in COVID-19 are scarce and need urgent attention of the scientific community, given the importance of this cytokine on inflammatory diseases (181). As already been reported, SARS-CoV increases this TNF-α production, leading to tissue damage (182). Moreover, the treatment with anti-TNF antibodies reduces the severity of lung disease for both influenza and respiratory syncytial virus (183), indicating that it could be efficient on COVID-19 cases.

Besides blocking such pro-inflammatory cytokines, the improvement of antiviral responses is also an interesting point to be considered. As earlier discussed, increasing evidence suggests obesity-associated impairment of IFN secretion, enhancing the susceptibility of this group for viral severe illnesses (73, 112). Reports have shown that IFN-β treatment reduces SARS-CoV RNA replication *in vitro* (184, 185). Indeed, type 1 IFNs are being pointed as potential effective therapeutic for COVID-19 and seems to be even more effective for SARS-CoV-2 compared to other coronaviruses (186). Moreover, given the impact of this cytokine for adaptive immune system activation, it might be suggested IFN also as a vaccine adjuvant for enhancing effective anti-viral protective response for especially individuals with obesity.

Obesity-induced chronic inflammation leads to alterations of hemodynamic properties and increasing risk for coagulopathies establishment. Moreover, the activation of coagulation pathway also triggers inflammation (167). Some recent case reports brought thromboembolism as a COVID-19 complication (187–189). Considering the interplay between coagulopathies and inflammation, the combination of anti-inflammatories and anticoagulants drugs could be key for avoiding systemic complications in COVID-19 patients with obesity. Heparin and its low-molecular derivate are examples of frequently used anti-coagulant drugs for tromboprophylaxis due to their inherent potential of preventing blood clot occurrence (190). Currently, heparin is also known for its anti-inflammatory properties, expanding its potential for the treatment of coagulopathies (191). Heparin is already recommended as prophylactic agent against thromboembolism for COVID-19 cases and preliminary data suggests a better prognosis after the treatment (192, 193); it is important to emphasize that heparin anti-inflammatory properties in addition to its anticoagulant function may explain its great potential compared to single target anticoagulant drugs (191). In addition, a report has shown that heparin also binds to the spike protein and partially inhibits SARS-CoV-2 invasion *in vitro*, thus presenting anti-viral potential (194). Besides, other anticoagulant drugs can also act on immune response, such as antithrombins and anti-factor Xa. Study the effectiveness of these drugs can be an important step for ameliorating complications that specially overweighed individuals are suffering by COVID-19 (167).

Chronic inflammation provided by obesity also intermediates the establishment of metabolic disorders. Diabetes and hypertension are considered a risk factor for developing SARS-CoV-2 severe illness, both in the presence and absence of obesity (160). The high mortality rate that affects these groups brought questions regarding the impact of daily medication on the viral infectiveness capacity. The use of angiotensin-converting enzyme inhibitors (ACEi) and angiotensin-receptor blockers (ARBs) seems to improve ACE2 expression on pulmonary cells (195). Increasing the number of the viral entry receptor could lead to higher severity of the disease. However, the available data about these modulations of humans' RAAS is too limited for conclusions (196). Until now, the impact of discontinuing this medication on individuals with diabetes or hypertension conditions can be much worse, due to protection of vital organs provided by them (197, 198). In addition, there is recent evidence that, in fact, those drugs could protect against COVID-19, once the virus leads to a reduction of ACE2; increasing the amount of this receptor could interfere on the viral pathway somehow (198). Moreover, ACE2 plays an important role on reducing inflammation, what could ameliorate complications of COVID-19 (199). A report has shown a reduction of IL-6 secretion and an improvement of antiviral immune response, decreasing the viral load (163). Regarding other medicines used for diabetes, such as metformin, no interaction was found with ACE2 expression, indicating that its intake should not be a concern (200). Analyzing the available data about anti-hypertensive and anti-diabetic drugs, it is clear that the harms related to their interaction with COVID-19 are not evidenced enough compared to the known risks of stopping the treatment. Thereby, the use of those medications may not be discouraged, once these untreated comorbidities highly increase the mortality risks for COVID-19, so as the risks of developing secondary health problems.

## Conclusion

A better understanding of the link between obesity and severe complications following COVID-19 infection is vital for implementing appropriate public health and therapeutic strategies to avoid COVID-19 severe symptoms and complications in people living with obesity. Adipose tissue from people with obesity show high expression of ACE2 receptor and can function as SARS-CoV-2 reservoir. Moreover, obesity can cause hyperglycemia via insulin resistance. SARS-CoV-2 may also cause hyperglycemia as well by infecting and killing pancreatic B-cells, leading to a worsen metabolic dysfunction of people with obesity and a poor prognostic of COVID-19. People with obesity present a pro-inflammatory and metabolic dysregulation that may favor the occurrence of the cytokine storm, implicated in COVID-19 pathophysiology of severe cases. Pulmonary lipofibroblasts can transdifferentiate into myofibroblasts aggravating the development of pulmonary fibrosis and consequently, contributing to the clinical severity of COVID-19, with development of a severe acute respiratory syndrome. Taken together, it may be crucial to better explore the role of visceral adipose tissue in the inflammatory response to SARS-CoV-2 infection and investigate the potential therapeutic effect of using specific anti-inflammatories (canakinumab or anakinra for IL-1β inhibition), anticoagulant (heparin), or anti-diabetic drugs in COVID-19 treatment since patients with obesity may benefit to a greater extent from a treatment that modulates these parameters.

## Author Contributions

GP-N, HB-M, SF, GK, and KM wrote different sections of the manuscript. KM revised, wrote, and prepared the manuscript. IS prepared the figures.

## Conflict of Interest

The authors declare that the research was conducted in the absence of any commercial or financial relationships that could be construed as a potential conflict of interest.
